# Immunogenic insights and experimental validation of a multiepitope vaccine construct against *Streptococcus pneumoniae*

**DOI:** 10.3389/fimmu.2026.1781081

**Published:** 2026-02-19

**Authors:** Yogeshwar Devarakonda, M. V. N. Janardhan Reddy, Arunima Binu, Kirtimaan Syal

**Affiliations:** Genetics and Molecular Microbiology Laboratory, Department of Biological Sciences, Birla Institute of Technology & Science, Pilani, Hyderabad, Telangana, India

**Keywords:** *Streptococcus pneumoniae*, multiepitope vaccine, immunogenic response, protein expression, cytokine profiling, antibody titers

## Abstract

*Streptococcus pneumoniae* is a predominant cause of pneumonia, sinusitis, bacteremia, meningitis and otitis media especially in children and the elderly. The emergence of antibiotic resistance and shift towards non-vaccine serotypes has compromised the efficacy of the treatment regime and available vaccines. The available polysaccharide-based vaccines confer protection against limited serotypes. In this study, we have conducted the experimental validation of a promising multi epitope vaccine candidate. The vaccine construct was cloned into pET-28a vector and expressed in *Escherichia coli*. The expression of the protein was induced, and its purification was carried out by the Ni-NTA affinity chromatography. Further, purified multiepitope protein was subjected to the SDS-PAGE and western blot analysis. The secondary structure was determined by circular dichroism spectroscopy, and it was observed to closely align with the predicted structure by Alpha fold. The hemolysis assay confirmed its safety and tolerability. The BALB/c mice was administered with this multiepitope vaccine construct and immune response was evaluated. The antibody response was estimated and confirmed by western blot and ELISA analysis. The cytokine profiling assay revealed the involvement of interleukins, TNF-α, and GM-CSF. Together, this study provides the first experimental validation of a computationally designed multi-epitope vaccine against *S. pneumoniae*, demonstrating antibody titers, broad cytokine response, and absence of hemolytic toxicity.

## 1 Introduction

*Streptococcus pneumoniae* is the major causative agent of pneumococcal infections and remains the leading infectious cause of morbidity and mortality worldwide. Before the discovery of antibiotics, it used to account for nearly 95% of pneumonia worldwide. According to UNICEF, the annual incidence rate of pneumonia in children has been observed to be 1 in 40 in South Asia and 1 in 62 in West and Central Africa respectively in 2024 (1). In 2019 alone, it caused nearly 740,180 deaths in children (2, 3). *S. pneumoniae* causes both invasive pneumococcal diseases such as meningitis and bacteremia, and non-invasive infections including sinusitis, otitis media, and acute bronchitis (4, 5). Evidently, over 100 distinct serotypes of *S. pneumoniae* have been identified globally. These serotypes are categorized based on the composition differences in their capsular polysaccharide (CPS) structure. The CPS plays a crucial role in providing protection against host immune responses, and therefore it serves as the principal target for pneumococcal vaccine development (6, 7). Recently, many non-vaccine serotypes such as serotype 38a have emerged, and majority of such serotypes remain uncharacterized (8, 9).

The PPV (pneumococcal polysaccharide vaccine) and PCVs (pneumococcal conjugate vaccine) are the commonly used vaccines against pneumonia (10). Both of the vaccines target CPS and induce serotype-specific immunity. Among the two, PCVs involve conjugation of these capsular polysaccharides to a protein carrier which potentially enable stronger T-cell dependent response (11). Evidently, despite the availability of these vaccines and gradual increase in vaccination coverage from 18.2% in 2018 to 23.6% in 2021, pneumococcal disease still remains a significant global health threat (12). While PPV23 covers a broader range of pneumococcal serotypes than PCVs, it is less immunogenic in infants and poorly effective in the elderly (13). In contrast, PCVs are highly effective, however they target only 10–13 serotypes. Moreover, the overall efficacy of both vaccines has been further compromised due to the emergence of non-vaccine serotypes, many of which also exhibit increased multidrug resistance (14, 15).

The protein based pneumococcal vaccines offer a cost-effective approach against non-typeable serotype and may also trigger serotype independent immune responses (16). Non-typeable serotype strains typically lack capsular layer but can still colonize and cause disease. These non-typeable strains are not targeted by current available capsular based pneumococcal vaccines. In recent years, several conserved cell surface proteins, which includes pneumococcal surface protein A (PspA), pneumolysin (PLY), and pneumococcal histidine triad protein D (PhtD) have been identified as promising targets for vaccine design (17–20). The approach for multi-epitope vaccine designs could combine immunodominant B- and T-cell epitopes from multiple antigens constituting a novel strategy that would potentially trigger broad and robust immune response (21).

Previously, we have designed a multiepitope vaccine construct against *S. pneumoniae* by the application of computational immunoinformatic tools (22). The epitope regions from outer cell wall proteins including Ply, PsaA, PspA, and PspK were selected and assembled in order to generate a multi-epitope vaccine (MEV) candidate. Various B and T cell epitopes within these proteins were predicted using bioinformatic tools. The designed MEV was assessed for antigenicity, allergenicity, toxicity, and other physicochemical properties through various *in-silico* methodologies including molecular simulation (22). However, experimental validation was not carried out earlier. In this work, we have focused on the experimental validation of the previously published *in silico* model of the MEV construct. The aim of this study is to evaluate the immunogenicity and safety of the vaccine through *in vitro* and *in vivo* analysis and compare the experimental results with the *in-silico* predictions.

## 2 Materials and methods

### 2.1 Bacterial strains and common chemicals

*Escherichia coli* XL1-Blue and BL21 (DE3) bacterial strains were employed for plasmid isolation and recombinant protein expression, respectively. Transformed cells were grown in Luria-Bertani (LB) medium with kanamycin at a concentration of 25 µg/mL. Ni-NTA resins were purchased from Qiagen, and antibodies used for western blot analysis were sourced from Thermo Fisher Scientific.

### 2.2 Cloning and expression of MEV construct

The amino acid sequence of the MEV construct was codon optimized for expression in *E. coli* system using the Twist bioscience (www.twistbioscience.com) online tool. The resulting 926 bp gene fragment was subsequently inserted into pET-28a plasmid at NcoI and XhoI sites. The insertion was structured to retain the C-terminal 6×His tag encoded in the vector. The final recombinant plasmid was constructed and outsourced from Twist biosciences.

The recombinant plasmid was introduced into *E. coli* BL21 (DE3) expression strain through the heat shock method. Positive colonies were cultured in LB broth along with kanamycin and incubated overnight at 37°C. Secondary cultures, inoculated from the primary culture, were grown to an OD_600_ of 0.6, followed by 0.5 ;mM IPTG induction. The cells were then pelleted by centrifugation and kept frozen at -20°C until further use.

### 2.3 Purification, refolding and confirmation

The recombinant MEV protein was purified through Nickel nitrilotriacetic acid (Ni-NTA) affinity chromatography. In brief, bacterial cell pellets from induced cultures were lysed under denaturing conditions, where lysis buffer along with 8 M urea was added. After sonication and centrifugation, the resulting supernatant was loaded onto a pre-equilibrated Ni-NTA column for binding. The beads were washed with low imidazole concentration (20 mM), then eluted with 300 mM imidazole. The urea-containing eluted protein was refolded using a stepwise dialysis method, where the eluted protein fraction was dialyzed against lower concentrations of urea, followed by a final urea-free dialysis with PBS buffer. The final refolded purified protein was resolved on 12% SDS-PAGE gel and visualized using Coomassie staining. Bradford protein micro-assay was performed to determine the concentration of MEV protein (23).

The MEV expression was confirmed using western blot analysis. The protein was resuspended in PBS buffer, and bands were resolved on SDS-PAGE, and bands were electro-transferred onto a PVDF membrane with BSA as the negative control. Mouse monoclonal anti-His antibody was employed as primary antibody, while a goat anti-mouse IgG conjugated with HRP was used as secondary antibody for detection. The protein bands were visualized by enhanced chemiluminescence (ECL), following the manufacturer’s protocol.

### 2.4 Structure analysis

The Circular dichroism (CD) spectrum of the MEV was recorded using Jasco J‐1500 spectropolarimeter and analyzed on Spectral manager II software. The Far-UV (260–195 nm) of MEV was recorded using a 1 mm path length cuvette with a scan speed of 50 nm/min. The mean of three individual scans was considered as observed spectra for further analysis. The obtained data was analyzed with the provided Spectral manager II software before plotting. The secondary structure composition prediction was calculated using BeStSel online tool (24). The tertiary three-dimensional structure was predicted with the application of AlphaFold 2 server. This predicted model was compared with the obtained experimental structure by performing secondary structure composition analysis.

### 2.5 Immunization of mice

A total of six female BALB/c mice aged six to eight weeks were randomly assigned into two experimental groups with three mice each. Group 1 served as the control and was administered phosphate-buffered saline (PBS) via intramuscular injection. In contrast, Group 2 was administered with 40µg of the MEV candidate. A booster dose identical to the primary immunization was administered on day 14 post-initial injection to increase the immune response.

The administration of the primary dose on day zero provides the initial exposure and stimulate primary immune response leading to the activation of immune cells (B and T cells) in addition to memory cells (25, 26). Blood collection on day 7 would capture this early primary immune response. Further, 7^th^ day blood sample provides baseline data to compare with post-booster immune response. The booster dosage administered on day 14 reinforces primary immune response triggering production of higher antibody titers and an enhanced cellular response (27, 28). The effect of booster dosage is typically evaluated on day 28 after the typical full vaccine schedule. Following the immunization schedule, blood samples were collected through the inner canthus of all mice at designated days post-immunization. Serum was separated from the blood sample through centrifugation, and serum samples were stored at -80 °C. The presence and specificity of antibodies generated against the MEV antigen were evaluated using western blot analysis (29).

### 2.6 Western blot analysis

Western blot was carried out to evaluate the reactivity of serum obtained from mice immunized with MEV antigen. The cell lysate of expressed protein MEV was resolved on SDS-PAGE gel and bands were further transferred onto a PVDF membrane. Following further blocking with skimmed milk, the anti-MEV serum obtained earlier was added to the membrane and incubated. The membranes were washed with TBS-Tween (TBST) thrice in order to prevent non-specific binding and later treated with secondary antibody (Goat anti-mouse IgG conjugated to HRP). The bands were observed using the ECL substrate solution (Bio-Rad) through a chemiluminescence imager.

### 2.7 ELISA (enzyme-linked immunosorbent assay)

Blood samples were collected from the inner canthus of mice on days 7 and 28 after immunization. The serum samples were subjected to indirect ELISA for assessment of antigen-specific antibodies. ELISA plate was coated with 0.2 μg per well of MEV overnight, blocked with BSA. After washing the wells with TBST, two-fold serial dilutions of serum were added. The wells were then washed and secondary antibody goat anti-mouse IgG HRP was added. After washing, TMB substrate was added and incubated in dark for 15 mins. The reaction was stopped by adding stop solution and the absorbance was measured at 450 nm using a microplate reader (30).

### 2.8 Bio-Plex Mouse Cytokine assay

The cytokine levels profiling of the serum samples was carried out using Bio-Plex Pro Mouse Cytokine Assay (Bio-Rad) kit. Briefly, magnetic beads were added to each well, followed by standards or samples. The plate containing the sample with beads was incubated at RT for 30 minutes with shaking and washed three times. Biotin-labeled detection antibodies were added and incubated for another 30 minutes. After washing, streptavidin-PE was added and incubated for 10–30 minutes. The beads were washed again and resuspended in assay buffer. The data was collected on the Bio-Plex 200 system, acquiring a minimum of 50 beads per analyte for each well, and subsequently analyzed using Bio-Plex Manager software with a five-parameter logistic (5-PL) curve.

### 2.9 Hemolysis assay followed by microscopy

Hemolysis of red blood cells (RBCs) in the presence of multi epitope vaccine construct was monitored. Blood sample obtained from the control mice was used for this study. Hemolysis assay was performed as described elsewhere (31). Briefly, the blood sample was centrifuged and the supernatant (plasma) was discarded. The resulting RBC pellet was washed and resuspended in PBS. Different protein concentrations (100 and 250 μg/mL) were incubated with diluted RBCs in a well plate. Positive and negative controls involved treatment with sodium dodecyl sulfate (20% SDS) and PBS, respectively. Samples were incubated and centrifuged to collect the supernatant. The absorbance was measured at 540 nm, each assay was performed in triplicates. The treated RBCs were examined under a light microscope (40x magnification) following slide preparation.

### 2.10 Statistical analysis

Statistical analysis was conducted using GraphPad Prism version 8. An ordinary One-way ANOVA followed by a multiple-comparisons test with the control group was employed to assess differences among multiple groups. Data are expressed as mean and p value <0.05 was deemed statistically significant.

## 3 Results

### 3.1 Expression and purification of MEV construct

**Figure 1:**
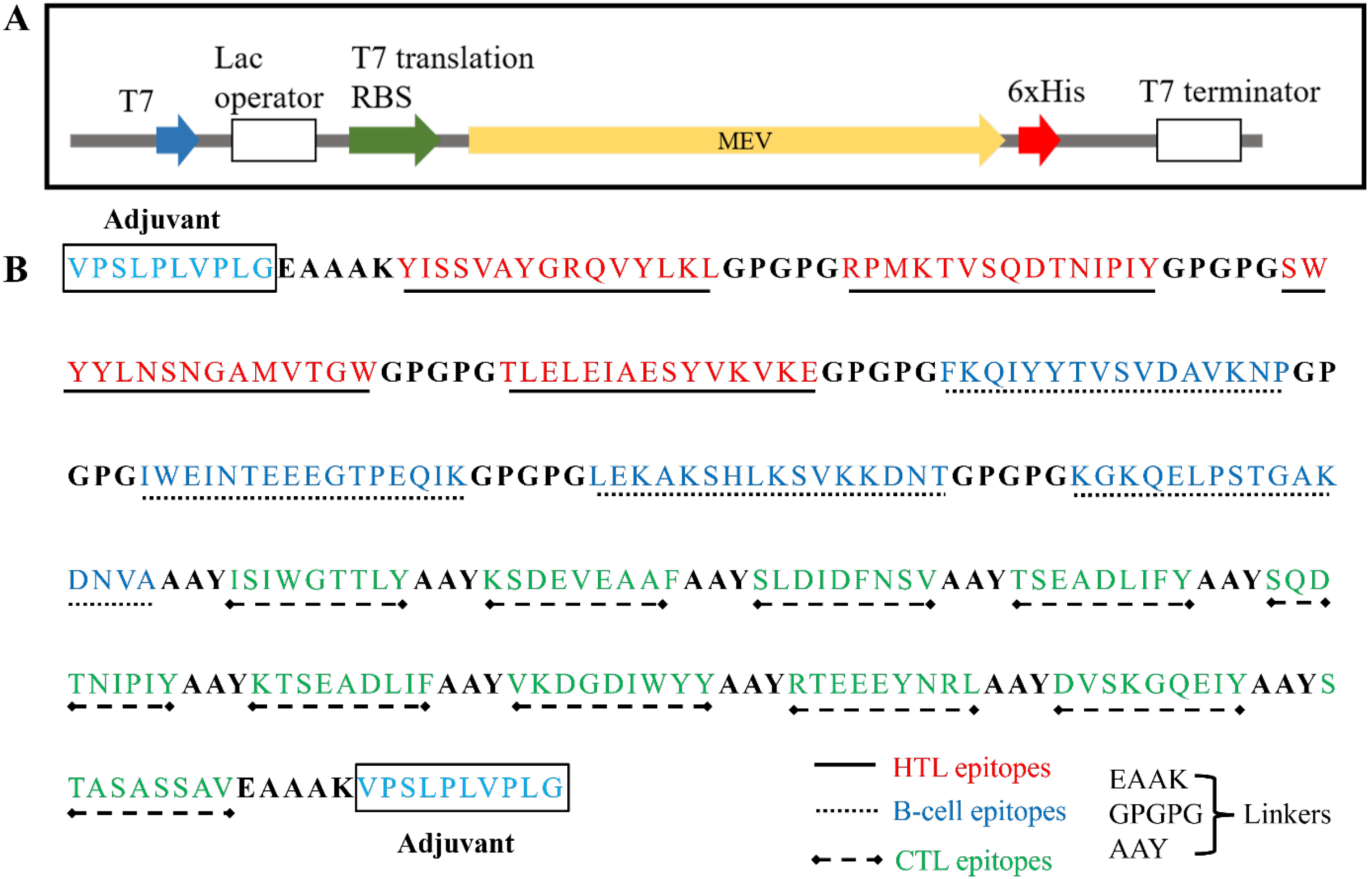
**(A)** Schematic representation of the open reading frame of MEV protein expression plasmid **(B)**. Amino acid sequence and features of the MEV construct containing HTL, B-cell, CTL epitopes along with linkers have been described.

The MEV protein was constructed using amino acid sequence derived from previously designed protein. The 927 bp MEV gene was synthesized after codon optimization to enhance the expression in *Escherichia coli* BL21(DE3). The optimized gene was successfully synthesized by Twist Bioscience (South San Francisco, CA, USA), and then it was cloned into pET28a vector, which included a 6×His tag at C-terminal for purification. This recombinant plasmid was successfully transformed into *E. coli* BL21(DE3) which was then used for the expression of MEV protein. The open reading frame and the insertion of MEV protein are shown in **Figure 1A**. Further, features of amino acid sequence of MEV construct have been described in **Figure 1B**.

**Figure 2:**
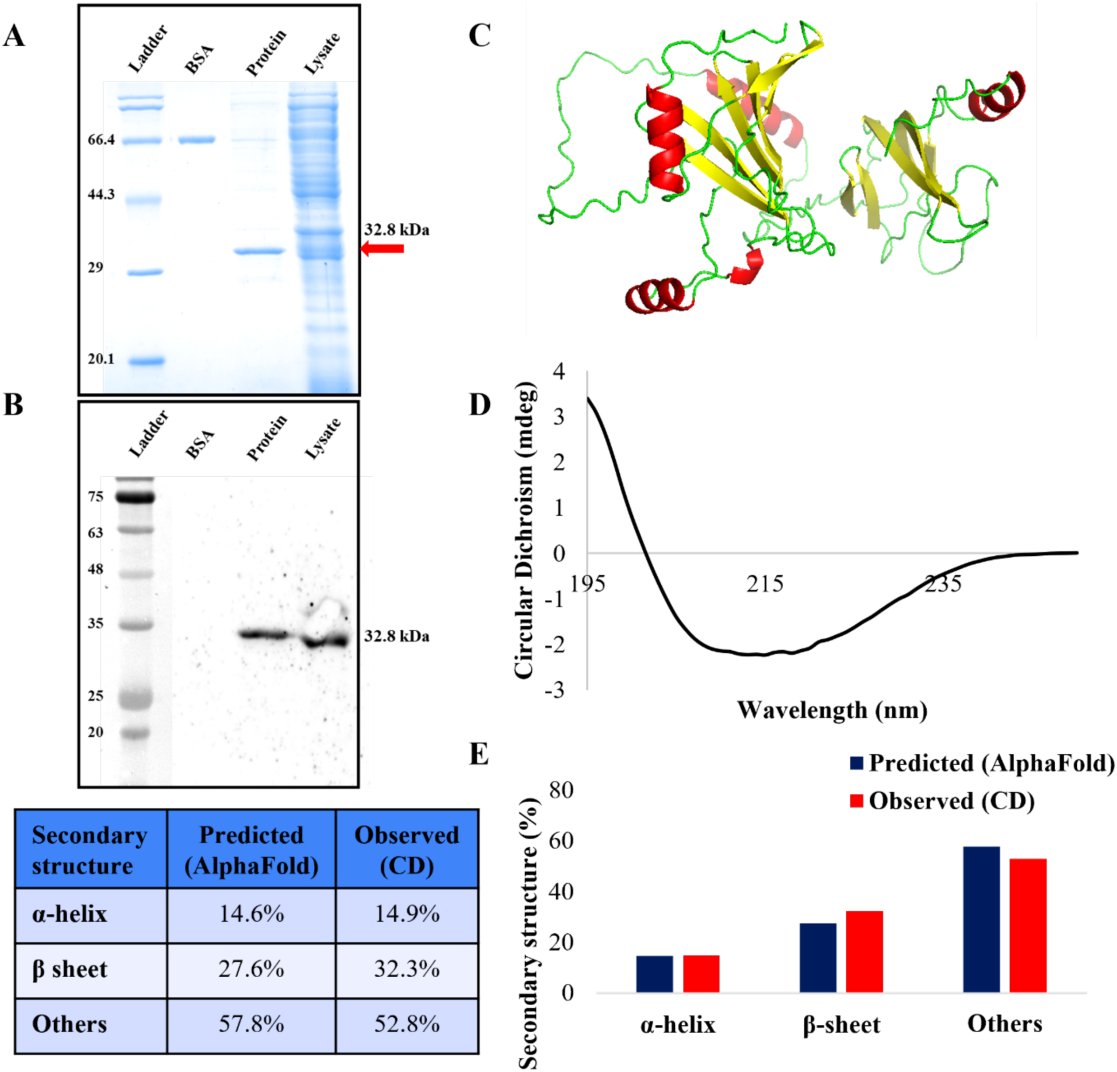
Expression, purification, western blot analysis and secondary structure composition of MEV protein. **(A)** Purified MEV protein along with BSA control has been analyzed by the SDS-PAGE gel analysis **(B)** Western blot analysis of MEV traced with anti-His antibody. **(C)** The 3D structure predicted by Alpha fold web tool. **(D)** CD spectrum of the MEV protein. **(E)** Bar graph showing quantitative secondary structure composition of predicted and experimental results. Table represented the predicted and observed secondary structure composition.

The MEV protein was overexpressed in *E. coli* cells. The T7 promoter system based pET28a plasmid featured C-terminal 6-His tag to enable affinity-based purification of the MEV protein expressed in *E. coli* system. The purification of the MEV protein was successfully carried out under denaturing conditions. The purity of MEV protein was confirmed by SDS-PAGE electrophoresis. The theoretical molecular weight of MEV protein was calculated through an online Expasy tool and was found to be 32.8 kDa. SDS-PAGE gel stained with Coomassie showed a protein band aligning with the expected molecular weight confirmed successful expression (**Figure 2A**). Western blot analysis with anti-His antibody verified the identity of purified protein, as shown in **Figure 2B**. Both the lysate and the purified protein along with BSA as negative control revealed a band corresponding to the His-tagged protein at approximately 32.8 kDa. These results validate the expression and purification of the MEV protein. The purified protein was further used for subsequent studies.

### 3.2 Secondary structure determination of MEV protein

The purified MEV protein was buffer exchanged to CD buffer (10mM potassium phosphate) to minimize the background interference before analysis. Far-UV CD spectroscopy was conducted to evaluate the secondary structural composition of the protein. This technique is commonly applied to assess the secondary structure of unknown proteins. The far-UV CD spectrum reflected the content of various secondary structures such as α-helix, β-sheet, and random coil. The far-UV CD spectrum data for the purified MEV protein was analyzed and quantified by the application of BeStSel which is an online tool for fold recognition and secondary structure determination based on CD spectra. The resulting far-UV CD spectra of protein (shown in **Figures 2C, D**) confirmed that the MEV protein retained its structure. The experimentally derived secondary structure of the MEV consisted of approximately 14.9% α-helices, 32.3% β-sheets and 52.8% other structures. This composition was further compared with the *in silico* secondary structure prediction through AlphaFold server, which showed highly similar values of 14.6% α-helices, 27.6% β-sheets, and 57.8% others respectively (**Figures 2C, E**). These results confirmed that the purified MEV protein has acquired a stable structure following purification.

### 3.3 Immunogenicity of MEV in mice

**Figure 3:**
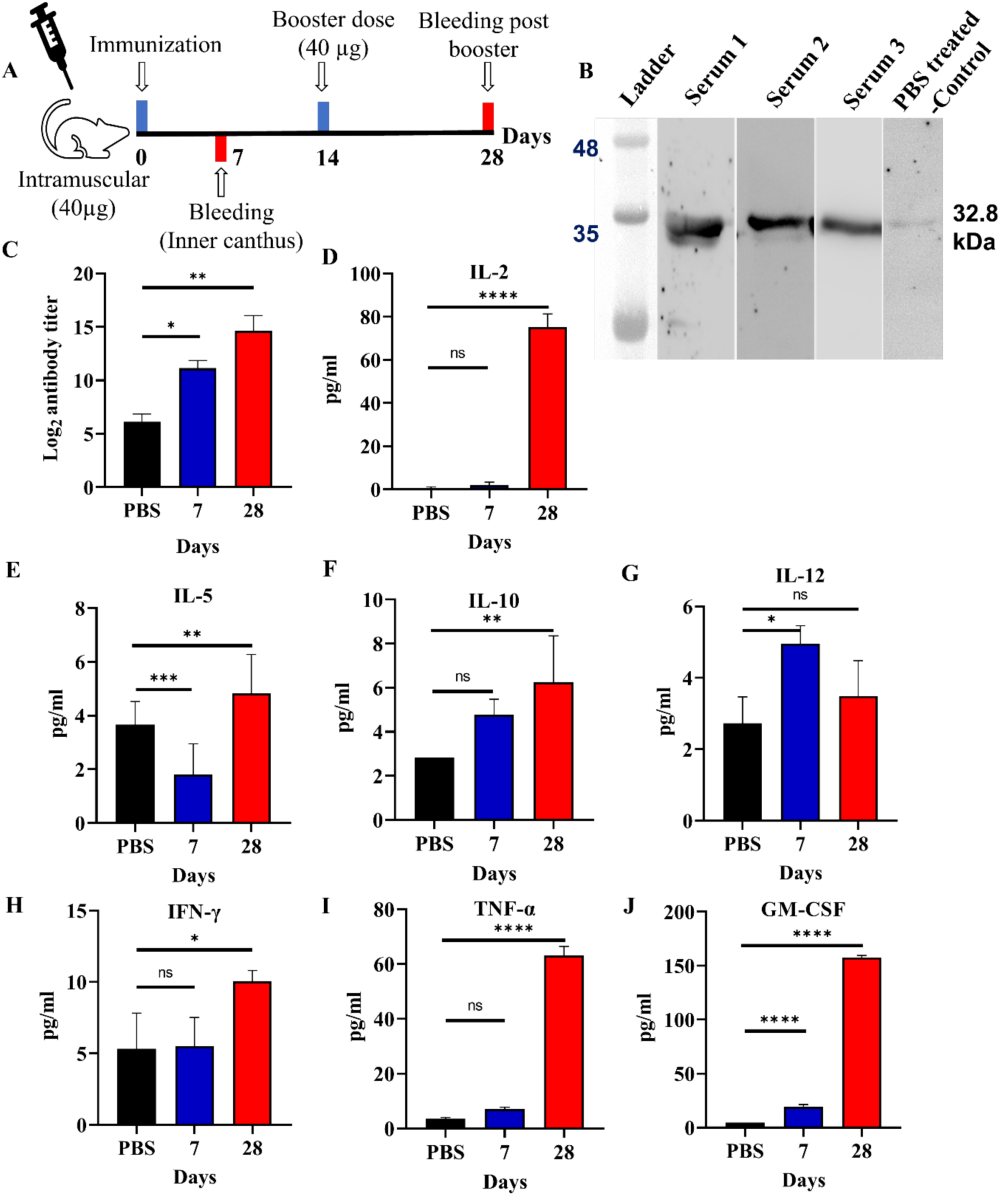
Immune response assessment. **(A)** Timeline depicting the active immunization schedule of the MEV vaccine, including the initial prime dose, 1 booster and 2 bleeding. **(B)** Western blot analysis showing the reactivity of anti-MEV serum against MEV protein after 28 days of immunization along with PBS-treated control serum sample. **(C)** Total IgG antibody titers of serum against MEV immunized mice- observed after 7 and 28 days of immunization where sera were tested with indirect ELISA. **(D-J)** Cytokine levels of IL-2, 5, 10, 12, IFN-γ, TNF-α, and GM-CSF were measured in mice post 7^th^, and 28^th^ days of immunization with MEV revealing increased concentrations on day 28 in comparison to negative control (PBS as placebo). Significantly different data as defined from One-way ANOVA is indicated by asterisks (p-values- * < 0.05, ** < 0.01, *** < 0.001, **** < 0.0001, ns- ‘not-significant’).

The immunization procedure for mice has been shown in the **Figure 3A**. Mice were immunized intramuscularly with 40 ;µg of antigen on day 0, followed by a booster dose on day 14. Blood samples were collected on days 7 and 28. Anti-MEV polyclonal antibodies in mice were confirmed through western blot analysis. A specific band was observed on PVDF membrane corresponding to the MEV protein band on SDS-PAGE gel upon western blot analysis which revealed a distinct band at approximately 32.8 ;kDa in all three replicates (Serum 1-3), confirming the presence of anti-MEV polyclonal antibodies (**Figure 3B**). The serum 1, 2, and 3 represent individual serum samples from three different mice in the vaccinated group (biological replicates) (**Figure 3B**).

### 3.4 Antibody titer in the anti-MEV serum of immunized mice

The collected serum from the inner canthus of mice at 7^th^ and 28^th^ day after immunization was tested for antigen-specific antibody through indirect ELISA assay. The endpoint titer refers to the highest serum dilution that could still elicit an immune response. Mice immunized with the MEV have showed significant immune response for 7^th^ and 28^th^ day serum samples in comparison to the control mice (**Figure 3C**).

### 3.5 Cytokine profile

The obtained results from the mice study showed a strong immune response especially on day 28^th^ after booster immunization. The levels of IL-2, TNF-α, and GM-CSF were significantly higher compared to the placebo group. GM-CSF exhibited the highest increase, indicating a plausible T cell activation and subsequent recruitment of myeloid cells. This suggests a Th1 immune response, which likely caused the observed effects. On day 28, IL-2, TNF-α, and GM-CSF levels were observed to increase in comparison to the control mice. IL-2 is known to promote the proliferation and growth of T cells, TNF-α plays a key role in mediating inflammatory responses, and GM-CSF helps in activation of granulocytes and macrophages. Cytokine analysis indicates a mixed Th1/Th2 response. Elevated IL-2, IFN-γ, TNF-α, and IL-12 suggest plausible activation of Th1-mediated cellular immunity, while increased IL-5 and IL-10 is suggestive of concurrent Th2 involvement. The sustained GM-CSF levels may further support enhanced antigen presentation and myeloid cell activation. (**Figures 3D–J**). Together, the immunization strategy suggests a humoral and cellular immune response against the MEV antigen, which would be further tested in a larger animal study coupled with T-cell profiling in near future.

### 3.6 Assessment of toxicity through hemolysis assay and microscopy

**Figure 4:**
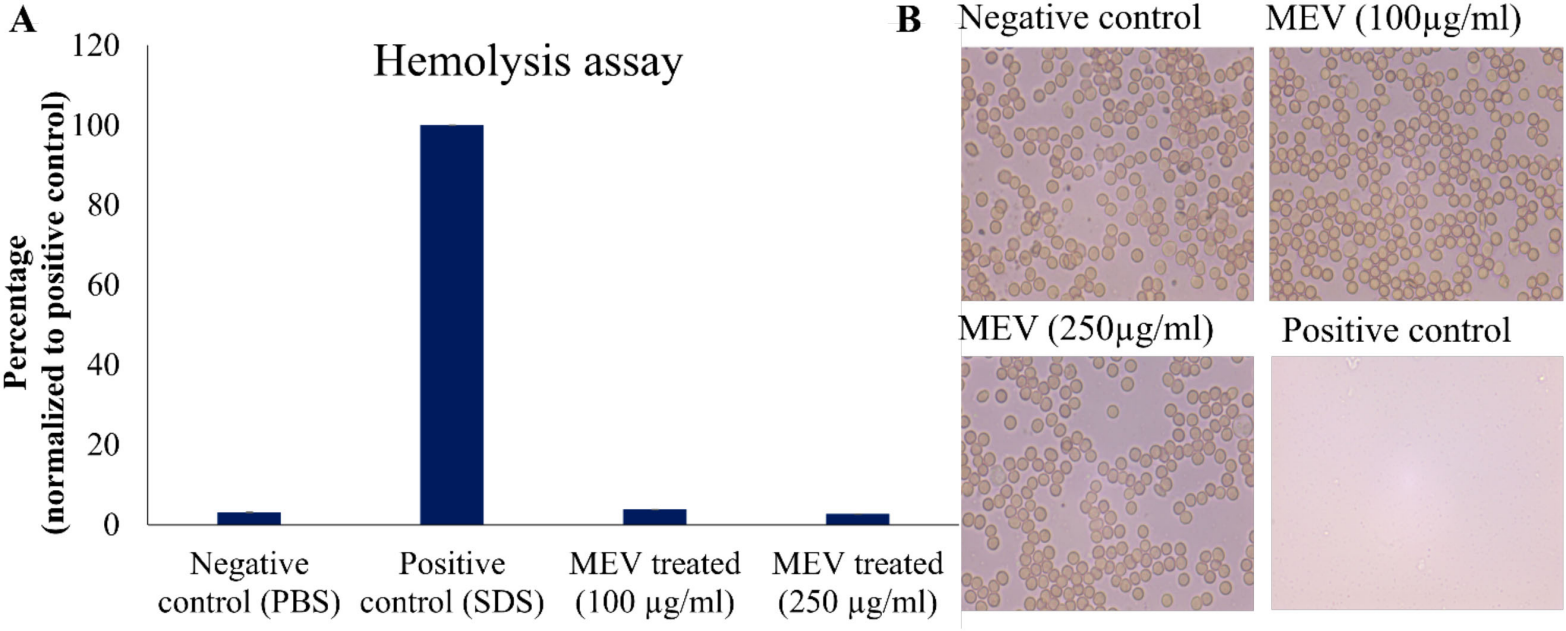
Toxicity assessment of MEV protein. **(A)** Percentage hemolysis of RBCs normalized to positive control. **(B)** Microscopic Analysis of RBC treated with 100, 250µg/mL MEV in comparison to the positive and negative controls.

The effects of MEV protein on RBCs have been evaluated by performing hemolysis assay and microscopy. The MEV protein at concentrations of 100 and 250 μg/mL was observed to be nontoxic to the RBCs. The results were comparable to the negative control where red blood cells were treated with PBS, as represented in **Figure 4A**. In contrast, SDS-treated cells served as the positive control and exhibited complete lysis. Treated and untreated cells were examined under 40x magnification using a microscope to assess any potential changes in morphology. Microscopic analysis revealed no change in cell morphology upon treatment with MEV and retained biconcave morphology (similar to negative control) as shown in **Figure 4B**.

## 4 Discussion

*S. pneumoniae* is the cause of pneumococcal diseases worldwide especially affecting the susceptible populations including children and elderly. Its pathogenicity and spread across the globe could be attributed to its capsular diversity, capacity to evade immune response, and the emergence of antibiotic resistance (8). *S. pneumoniae* may also reside asymptomatically in the upper respiratory tract and cause severe disease when immunity is compromised (8, 32). As described in earlier sections, the key shortcoming of currently available pneumococcal vaccines is their limited serotype coverage and the consequent emergence of non-vaccine serotypes (33).

The multi-epitope construct integrates protective epitopes from multiple pneumococcal antigens to enhance antigen presentation and immune response. This strategy aims to elicit a broader and effective immune response, potentially offering protection across a wide range of serotypes. Evidently, vaccines made up of a combination of multiple epitopes are known to elicit a better immune response in comparison to those based on a single antigenic component (34, 35). The multi-epitope vaccines are likely to stimulate B and T cell immune response more efficiently than vaccines based on the single full-length protein as the full length protein may not be required to trigger an immune response (36, 37). Multiepitope-based vaccines offer several advantages, including their capability to stimulate a targeted immune response against specific epitopes, thereby enhancing the overall effectiveness of the vaccine (38, 39).

Building upon our previous study, we have already designed and constructed an *in silico* multi-epitope vaccine candidate against *S. pneumonia* (22). In this study, the cloning, expression, and purification of the MEV construct in *E. coli* BL21 (DE3) were successful carried out, which also proved the effectiveness of codon optimization and vector design in increasing the yield of recombinant protein. The use of denaturing conditions and the subsequent refolding indicates that the MEV protein was mainly present in the insoluble form. This is a normal occurrence for the multi-epitope constructs or proteins with complex tertiary structures and also emphasizes the need for the optimization of refolding protocols in order to recover the functional protein (40, 41).

Although western blotting provides qualitative confirmation, quantitative studies including antibody sub-class profiling would be conducted in future studies. The experimental evaluation of the MEV protein by CD measurements suggest that the purified protein maintained a stable conformation, indicating proper folding and structural integrity. The comparative analysis of the CD spectra and *in silico* predicted results obtained from AlphaFold server for the protein showed high similarity with insignificant differences. However, CD and Alphafold2 assess the secondary structure and theoretical folding capacity, respectively. The pneumolysin neutralization assays, binding studies of immune sera with native pneumolysin/PspA and challenge experiments will be carried out in near future, which would further confirm the structural integrity of different epitopes in multiepitope construct. It is noteworthy that the MEV construct retained immunogenicity despite undergoing denaturation and refolding suggesting successful recovery of conformational epitopes.

The specific antibodies in the serum induced by MEV could detect MEV protein, indicating a successful induction of an antigen-specific humoral immune response. The indirect ELISA results showed a higher titer of antigen-specific antibody in the 7- and 28-day serum samples of mice immunized with MEV protein. These results suggest that the MEV immunized mice were capable of inducing humoral response after the booster dose. Notably, the levels of cytokines IL-2, IFN-γ, TNF-α, and GM-CSF increased significantly, suggesting T-cell activation and myeloid cell recruitment whereas the increased levels of IL-5 and IL-10 suggests activation of humoral and regulatory immune response. MEV was observed to be non-toxic to RBCs as validated by hemolysis and microscopy-based assays. The animal experiment was designed as a pilot study with an aim to generate preliminary data on immunogenicity and safety. Since, this pilot study showed potential, it will be conducted in larger animal cohorts for validation in near future.

Together, our findings suggest that the MEV protein is immunogenic and capable of eliciting a broad immune response supporting its potential as a promising candidate for future vaccine development and therapeutic applications. However, it requires further validation by challenge experiments and detailed assessment of immune response including cytokine profiling and antibody typing. Also, we propose to conjugate the proposed multi-epitope vaccine construct with polysaccharides for the development of serotype-independent pneumococcal vaccine that could also protect against non-typeable strains in near future.

## Data Availability

The original contributions presented in the study are included in the article/supplementary material. Further inquiries can be directed to the corresponding author.
